# Supplementary material for: Ethylene Plays a Dual Role during Infection by Plasmodiophora brassicae of Arabidopsis thaliana
Source: Genes (Basel). 2022 Jul 22;13(8):1299. doi: 10.3390/genes13081299 (PMC9329982; doi:10.3390/genes13081299)
Supplement: Supplementary file 1 [file genes-13-01299-s001.zip › genes-1816661-supplementary.pdf]

Table S1 Primers used for mutant identification and qRT-PCR

Name	Sequence (5'-3')	Description	Gene ID
AtEIN3 -F	CTGCAGATCACAACAACTTTGA	q-PCR	AT3G20770
AtEIN3 -R	CATCCATCGTTCCTACTACTCC	q-PCR	
AtEIL1 -F	TTGAAGAAAGCTTGGAAAGTCG	q-PCR	AT2G27050
AtEIL1 -R	TTTGATTGCCTCACAAGCTTAC	q-PCR	
AtSHN1 -F	CCACAATGTCATCCTCAACATC	q-PCR	AT1G15360
AtSHN1 -R	GCGTCTTGTGAAGCTTTACTAG	q-PCR	
AtWRKY75 -F	ATCAGAAGGTTGTTCGAAAAGC	q-PCR	AT5G13080
AtWRKY75 -R	CCTCCATCGATAACCATCATCA	q-PCR	
At2G20350 -F	GTCTCTGCTTTGCAACAAGTAA	q-PCR	AT2G20350
At2G20350 -R	GCCCATTTACCTGATGGTTTTT	q-PCR	
AtERF105 -F	ACAAGGCAGCTTTTAAACTACG	q-PCR	AT5G51190
AtERF105 -R	TGAGCTTCTTCCCTCTTAACAG	q-PCR	
AtWRKY45 -F	GCCAGGTTGATATACTGGATGA	q-PCR	AT3G01970
AtWRKY45 -R	TGCACTTATAATAGCTCCTGGG	q-PCR	
AtRALFL1 -F	GGAGGAAGAAGAAATGGACTCA	q-PCR	AT1G02900
AtRALFL1 -R	TCCGTTTCAAAGACTGATAGCT	q-PCR	
AtLRX10 -F	GTTGATTTGAATGGTGCGGATA	q-PCR	AT2G15880
AtLRX10 -R	TTAGGCTCAACTTCTCGAAACT	q-PCR	
AtFER -F	CTATCGAAGACTGGTCCTACAC	q-PCR	AT3G51550
AtFER -R	ATAGAACAACGCCAAAGGAGTA	q-PCR	
AtRALFL33 -F	TCTGAGATCAACAGGCGTATTT	q-PCR	AT4G15800
AtRALFL33 -R	CGTCGGCAATTGTAGTAAGATG	q-PCR	
AtRALFL22 -F	ACTTCGGAGATTCGCTAGATTT	q-PCR	AT3G05490
AtRALFL22 -R	CCATCTCTTCTTCTTCCGCTAT	q-PCR	
AtRALF23 -F	GAGATGGACTCAGAGATCAACC	q-PCR	AT3G16570
AtRALF23 -R	GACAATTGTAGTAAGATGCGCC	q-PCR	
AtRALFL24 -F	GTAATCGGAGAGGAAGAAGGAG	q-PCR	AT3G23805
AtRALFL24 -R	AGTGTCTCTAGCACACCTAGTA	q-PCR	
AtRALFL27 -F	CTTGTGATGGTAGAATTGCTGG	q-PCR	AT3G29780
AtRALFL27 -R	ACGTTTGCATCTCTGGTAGTAA	q-PCR	
AtLRX3 -F	TCAGTTACCGAAGCTTGAGAAT	q-PCR	AT4G13340
AtLRX3 -R	GACAAAAACGCTTTACATTGCC	q-PCR	
AtRALFL31 -F	GCAATGTTGATCCGAAACAGTA	q-PCR	AT4G13950
AtRALFL31 -R	GTTTCTGACAAGGAACCATGTC	q-PCR	
AtRALFL32 -F	GAGACAGAGGAAATGAGTGTGA	q-PCR	AT4G14010
AtRALFL32 -R	CTCCATACGAAAGCTTATGTGC	q-PCR	
AtLRX5 -F	CGGTTGAAAAACGTAACGGTAT	q-PCR	AT4G18670
AtLRX5 -R	AGAGAATCTATTATGCGCCACA	q-PCR	
AtRALFL34 -F	GAAGAAAGCTTCGAAGTTACGG	q-PCR	AT5G67070
AtRALFL34 -R	TATGTAATACTTCGTCCTCCGC	q-PCR	
AtTHI2.1-F	GCAAGTGAAATTGTGAATG	q-PCR	AT1G72260
AtTHI2.1-R	CACACACACACACAAACCAGAA	q-PCR	
AtARGAH2-F	TGGTACTTTGGAGTTTAATCGTTG	q-PCR	AT4G08870
AtARGAH2-R	TGTCTATGAGACCACACTTATTGC	q-PCR	
AtACTIN2 -F	TGTGCCAATCTACGAGGGTTT	q-PCR	AT3G18780
AtACTIN2 -R	TTTCCCGCTCTGCTGTTGT	q-PCR	
AtPR2 -F	CGTTGTGGCTCTTTACAAACAA	q-PCR	AT3G57260
AtPR2 -R	AGCTCTGAACGTTTTCTTGAAC	q-PCR	
wrky75-c -F1	ATTCAGGTGGATCGGTCTGT		
wrky75-c -R1	GCCATATTTCCTCCATCGATAACC	identification of target 1	
wrky75-c -F2	GGATTCGGGAACATATATGTGACA		
wrky75-c -R2	GCATTTGAGTGAGAATATGCTCG	identification of target 2	
EIN3 -F1	CGCTTTTGTCTGCGTTGAT	identification of *ein3*	
EIN3 -R1	ATAGCAAGCCAGGTAGCAC		
EIL1 -F1	GAGAGAGATACCACTTTTGGTG	identification of *eil1*	
EIL1 -R	CACAGGCTTACCCTTCTCAG		
